# Weight Gain in Infancy and Childhood Were Associated With Pubertal Development in Boys and Girls

**DOI:** 10.1111/apa.70148

**Published:** 2025-05-23

**Authors:** Saga Rebecca Nummela, Katja Pahkala, Sinikka Karppinen, Jorma Toppari, Olli Raitakari, Harri Niinikoski

**Affiliations:** ^1^ University of Turku Turku Finland; ^2^ The Wellbeing Service County of Southwest Finland Turku Finland; ^3^ Centre for Population Health Research University of Turku and Turku University Hospital Turku Finland; ^4^ Research Centre of Applied and Preventive Cardiovascular Medicine University of Turku Turku Finland; ^5^ Paavo Nurmi Centre, Unit of Health and Physical Activity University of Turku Turku Finland; ^6^ Department of Pediatrics and Adolescent Medicine Turku University Hospital and University of Turku Turku Finland; ^7^ InFLAMES Research Flagship University of Turku Turku Finland; ^8^ Institute of Biomedicine, Research Centre for Integrative Physiology and Pharmacology University of Turku Turku Finland; ^9^ Department of Pediatrics Turku University Hospital Turku Finland; ^10^ Department of Clinical Physiology and Nuclear Medicine Turku University Hospital Turku Finland

**Keywords:** cardiometabolic risk, childhood, infancy, puberty, weight gain

## Abstract

**Aim:**

As earlier puberty has been associated with higher later metabolic risk, we studied how weight gain in infancy and childhood is associated with pubertal timing and duration in healthy children.

**Method:**

Leveraging the longitudinal data from the Special Turku Coronary Risk Factor Intervention Project study, we used linear regression analyses to investigate how weight gain in different age intervals during infancy and childhood is associated with the onset, culmination and duration of puberty in 230 boys and 278 girls.

**Results:**

For girls, a 1 standard deviation (SD) greater weight gain in infancy and childhood predicted earlier onset of breast development and earlier menarche by approximately 3–4 months. For boys, a 1 SD greater weight gain in infancy and childhood predicted earlier start and culmination of genital development according to Tanner stages by approximately 2 months. Greater weight gain in infancy and childhood seems to lengthen the duration of puberty in girls by 1–2 months, but is not associated with the duration of puberty in boys.

**Conclusions:**

Weight gain in infancy and childhood was associated with the timing of puberty in both boys and girls.

**Summary**

- The onset of puberty is currently earlier than before, and earlier puberty has been associated with a higher metabolic risk later in life.
- Greater weight gain during infancy and childhood predisposes to earlier onset and culmination of puberty in boys and girls and longer duration of puberty in girls.
- These data improve our understanding of the associations between early weight gain and pubertal timing.

**Abbreviations**
AGA: appropriate for gestational age
BMI: body mass index (BMI)
CI: confidence interval
SD: standard deviation
SDS: standard deviation score
STRIP: Special Turku Coronary Risk Factor Intervention Project
T ≥ 25 mm: first testes measurement ≥25 mm

## 1 Introduction

Boys and girls are going through puberty earlier than in past decades [1, 2]. As an independent factor, earlier puberty has been associated with a higher prevalence of cardiometabolic risk factors, including overweight and obesity, dyslipidaemias, higher glucose levels and endocrine‐related cancers [3, 4]. Furthermore, earlier puberty may increase the risk for psychosocial difficulties, such as depressive and anxiety disorders, as well as lower educational outcomes [5].

The incidence of childhood obesity has also been increasing [1, 6]. Childhood overweight and obesity have clearly been associated with earlier onset of puberty in girls. Overweight in boys has been associated with earlier pubertal onset, whilst obesity has been found to possibly delay pubertal onset [7]. Of note is that the findings for boys are, in general, much less convincing than for girls [7, 8, 9]. Several studies have attempted to map out whether growth or increased body mass index (BMI) during a specific time in infancy or childhood could be particularly significant for the age of pubertal onset. The results have been conflicting, some finding early and others late infancy to be of more significance [10, 11, 12]. Height development in infancy, childhood and puberty is regulated differently, and therefore, it is good to investigate these growth periods separately [13].

For girls, puberty begins with the start of breast growth, defined with Tanner stage M2, and culminates in menarche, which takes place near the end of pubertal development [14]. The age at the time of menarche is an easily identified event, while there is no equivalent event in boys. Male pubertal development has begun when testicular size is ≥ 25 mm in length, corresponding to 3 mL, and ends in Tanner stage G5 [15, 16].

Despite increasing research on the links between early growth and pubertal timing, the duration of the sexual maturation process has seldom been the focus of interest. Thus, there is a lack of knowledge on pubertal timing and duration in combination with data on infancy and childhood growth patterns, especially in normal weight children born appropriate for gestational age (AGA) [17, 18]. In addition, the impact of body composition on pubertal development is often studied with the prerequisite of obesity in the study population [8].

Considering the previous contradicting findings and gaps in knowledge, there is an evident need for more in‐depth research, especially on the associations of early growth with the timing and duration of puberty in healthy children. However, prospective data sets with pubertal milestones measured and not self‐reported that offer the opportunity to shed light on the topic are scarce. The aim of this study was to investigate how the development of weight standard deviation scores (SDS) in different age intervals in infancy and childhood was associated with the onset, culmination and duration of puberty in both boys and girls. We did this by using the unique longitudinal data from the Special Turku Coronary Risk Factor Intervention Project (STRIP).

## 2 Methods

### 2.1 STRIP Study Design

STRIP is a prospective, randomised, controlled infancy‐onset trial aiming to prevent atherosclerosis risk factors. The recruitment of 5‐month‐old infants and their families at well‐baby clinics in Turku, Finland, took place between early March 1990 and June 1992. Initially, 1880 families responded to the study invitation, and 1105 families were interested in participating. The final study comprised 1062 infants and their families who were randomly allocated into the intervention group (*n* = 540) or control group (*n* = 522) [19].

The intervention group received individualised dietary counselling twice a year, beginning at 8 months of age. This dietary counselling was based on the Nordic Nutrition Recommendation available at that time, and the main aim of the intervention was to replace saturated fat with unsaturated fat. Decreasing total fat consumption was not the aim. A secondary aim of the intervention was to increase intake of fruits, vegetables and whole‐grain products. The control group was observed twice a year up to their seventh birthday and then annually until 20 years of age. The control group was observed once a year until 20 years of age and received basic health education given at Finnish well‐baby clinics and school health care. Both study groups met the same study personnel, and similar measurements were performed on both groups.

In the present study, the groups were combined, as we had previously shown that pubertal development of the intervention and control groups was similar [20]. This current research paper included all study participants of whom growth and pubertal information was available, with the following exclusions. First, participants with known conditions that affect growth and/or pubertal development were excluded from the present analyses. These were 5 individuals with Down syndrome, cerebral palsy and other developmental disorders. Second, 26 girls were excluded due to inaccuracies in reported menarche and observation of M2, where the pubertal duration came out to be < 0. Likewise, 3 boys whose pubertal duration was reported to be < 1 year due to inaccuracies in observation of testicular development were excluded. Third, because of the previous contradicting findings of the impact of overweight versus obesity on pubertal development in boys [7, 9], we chose to exclude the 5 subjects who were classified as obese at the first pubertal milestone [16]. Finally, after 39 exclusions and 515 children lost to follow‐up, data of 278 females and 230 males were used. Most of these participants were AGA (96% of girls and 95% of boys). However, 1.6% of the girls and 1.3% of the boys were small for gestational age (< −2 standard deviations (SD)) and 2.7% of the girls and 3.9% of the boys were large for gestational age (> +2 SD). There were two twin pairs in the study.

### 2.2 Measurements of Growth and Pubertal Stage

The first measurements of length and weight were obtained at 7 months. Measurements before that were obtained from databases maintained by maternity hospitals and well‐baby clinics. Further measurements were carried out at 13 and 18 months and then annually from 2 years onwards. Before the age of 18 months, weight was measured with an infant scale (model 725; Seca, Murrhardt, Germany); measurement of weight from the age of 2 years onwards was made with an electronic scale (S10; Soehnle, Murrhardt, Germany) to the nearest 0.1 kg. Length was measured before the age of 2 years with an infant board (Bekvil; Paljerakenne, Helsinki, Finland), and after 2 years of age, height was measured to the nearest 0.1 cm with a Harpenden stadiometer (Holtain, Crymych, UK).

Pubertal stages were monitored from the age of 9 years using Tanner staging [16]. Breast development (Tanner M) in girls was evaluated by palpation and measurement of breast tissue using a ruler. Similarly, the pubertal stage of external genitalia in boys (Tanner G) was determined visually and by measuring the length of the testes with a ruler. The age of menarche was self‐reported by study participants with an accuracy of a month [21, 22]. Measurements obtained at annual study visits were used for both intervention and control participants. The first pubertal milestone was set for girls as the first measurement of Tanner staging >M1 and for boys as the first measurement of testes ≥ 25 mm in length (T ≥ 25 mm) at the annual study visit. The end of puberty was defined as the age of menarche for girls and the age of the first measurement of G5 for boys. The duration of puberty was the difference between pubertal milestones.

### 2.3 Statistical Analyses

Distribution of the continuous variables was evaluated visually and using the Kolmogorov‐Smirnov test. Normally distributed variables were presented using mean values and standard deviations (SD).

Growth was evaluated as weight gain in different age intervals: birth to 7 months, 7–13 months, 13–18 months, 18–24 months, birth to 13 months, birth to 24 months, 2–3 years, 3–4 years, 4–5 years, 5–6 years, 6–7 years, 7–8 years and 2–8 years. Birth to 24 months represented growth during infancy, and 2–8 years represented growth during childhood [23]. Weight gain within all age intervals was normally distributed (Table S1). Weight gain in different age intervals was converted to SDS for analyses. Additionally, growth was assessed using height development converted to SDS of height in all the same age intervals. Height development was normally distributed in all age intervals (Table S2).

Associations between weight gain in different age intervals and puberty milestones and duration of puberty were studied using linear regression analyses. Linear regression analyses were made with no adjustments and then by adjusting for BMI at the pubertal milestone in question and for the duration of puberty with the BMI at the first pubertal milestone. Similarly, the association of height development in different age intervals with puberty milestones and duration of puberty was studied using non‐adjusted linear regression analyses.

All statistical analyses were conducted using SAS statistical software, release 9.4 (SAS institute inc., North Carolina, USA). For results, *p*‐values < 0.5 were considered statistically significant, and results also present 95% confidence interval (CI).

### 2.4 Ethics

Informed consent was obtained from the parents at the beginning of the study and from the children at the ages of 15 and 18 years. The study protocol of the STRIP Study was approved by the local ethics committee on 7 November 1989, and the guidelines of the Declaration of Helsinki were followed [24].

## 3 Results

The mean age for >M1 in girls was 11.1 (SD 1.3) years, and the age for T ≥ 25 mm in boys 12.2 (SD 1.0) years. The mean age of menarche was 13.0 (SD 1.2) years, and the mean age of G5 was 16.1 (SD 1.1) years in boys. The duration from >M1 to menarche was an average of 2.0 (SD 1.1) years, whilst among boys the duration from T ≥ 25 mm to G5 was 3.9 (SD 1.2) years (Table 1).

**TABLE 1:** Ages at puberty milestones and puberty duration in girls and boys.

	Age at Tanner >M1/T ≥ 25 mm (years)	Age at menarche/Tanner G5 (years)	Puberty duration^a^ (years)
	Mean, (SD)	Mean, (SD)	Mean, (SD)
Girls (*n* = 278)	10.99 (1.27)	12.95 (1.21)	1.96 (1.10)
Boys (*n* = 230)	12.23 (1.0)	16.12 (1.07)	3.89 (1.21)

^a^ > M1 to Menarche in girls, T ≥ 25 mm to Tanner G5 in boys.

Adjusting for birth weight did not affect the results, which is why no analyses were adjusted for it.

### 3.1 Girls

Weight gain and age at start of puberty (Tanner >M1) were inversely associated in all age intervals from the 18–24‐month interval onwards as well as in the intervals describing infancy growth (birth to 13 months, birth to 24 months) and childhood growth (2–8 years) (Table 2). Similarly, an inverse association was found between age of menarche and weight gain in the birth to 2‐year interval, and from the 2–3‐year interval onwards, as well as in the childhood growth interval 2–8 years. The strongest associations were observed in the 2–8‐year interval and in year‐long intervals. The strongest associations were observed between weight gain at the 6–7‐year interval and the pubertal milestones. A 1 SD increase in weight gain during the 6–7‐year interval corresponded with 4.2 months (= 0.35 year, CI 0.26 to 0.43, *p*‐value < 0.0001) earlier age at >M1 and with 3.5 months (= 0.29 years, CI 0.19 to 0.38, *p*‐value < 0.0001) earlier age at menarche. Furthermore, weight gain was directly associated with the duration of puberty from the 4–5 year age interval onwards and with the 2–8‐year interval, indicating that increased early weight gain is linked with a longer duration of puberty (Table 2).

**TABLE 2:** Associations of weight gain (standard deviation score, *z*‐score) with the youngest age of Tanner stage > M1, age of menarche and duration of puberty in girls.

	Age at Tanner >M1 (years)	*p*	Age at menarche (years)	*p*	Duration of puberty^a^ (years)	*p*
	Beta^b^ CI		Beta^b^ CI		Beta^b^ CI	
Birth to 7 months	−0.09 −0.18 to 0.004	0.06	−0.09 −0.18 to 0.01	0.07	0.01 −0.09 to 0.11	0.86
7 to 13 months	−0.04 −0.13 to 0.06	0.46	−0.02 −0.12 to 0.09	0.76	0.03 −0.08 to 0.14	0.60
13 to 18 months	−0.05 −0.14 to 0.04	0.28	−0.04 0.14 to 0.06	0.45	0.02 −0.08 to 0.13	0.65
18 to 24 months	**−0.12** **−0.22 to − 0.03**	**0.01**	−0.05 −0.16 to 0.05	0.33	0.11 −0.01 to 0.22	0.07
Birth to 13 months	**−0.11** **−0.20 to − 0.02**	**0.02**	−0.10 −0.19 to 0.001	0.05	0.03 −0.08 to 0.14	0.57
Birth to 24 months	**−0.15** **−0.24 to − 0.06**	**0.002**	**−0.12** **−0.22 to − 0.02**	**0.02**	0.06 −0.05 to 0.17	0.29
13 to 24 months	**−0.013** **−0.22 to − 0.03**	**0.01**	0.09 −0.19 to 0.01	0.09	0.06 −0.04 to 0.17	0.25
2 to 3 years	**−0.20** **−0.29 to − 0.11**	**< 0.001**	**−0.15** **−0.25 to − 0.06**	**0.002**	0.08 −0.03 to 0.19	0.14
3 to 4 years	**−0.17** **−0.26 to − 0.08**	**< 0.001**	**−0.13** **−0.23 to − 0.04**	**0.01**	0.07 −0.04 to 0.18	0.20
4 to 5 years	**−0.27** **−0.36 to − 0.18**	**< 0.001**	**−0.16** **−0.25 to − 0.06**	**0.002**	**0.17** **0.07 to 0.28**	**0.002**
5 to 6 years	**−0.35** **−0.43 to − 0.26**	**< 0.001**	**−0.25** **−0.34 to − 0.15**	**< 0.001**	**0.17** **0.06 to 0.27**	**0.003**
6 to 7 years	**−0.35** **−0.43 to − 0.26**	**< 0.001**	**−0.29** **−0.38 to − 0.19**	**< 0.001**	**0.11** **0.0001 to 0.22**	**< 0.05**
7 to 8 years	**−0.28** **−0.37 to − 0.18**	**< 0.001**	**−0.16** **−0.26 to − 0.06**	**0.002**	**0.17** **0.06 to 0.28**	**0.003**
2 to 8 years	**−0.43** **−0.51 to − 0.35**	**< 0.001**	**−0.30** **−0.40 to − 0.21**	**< 0.001**	**0.21** **0.10 to 0.32**	**< 0.001**

*Note:* Bold indicates *p* < 0.05.

^a^ >M1 to Menarche in girls.

^b^ The beta estimate represents the association of 1 SD increase in growth with the outcome variable in years (Beta × 12 gives the estimate in months). A negative value describes earlier pubertal milestone/shorter duration of puberty.

Assessing growth using height development, the results were quite similar to weight gain. The inverse associations between growth in height and the puberty milestones were significant in many of the age intervals: for Tanner stage >M1 from the 1–2 ‐year age interval onwards and in the birth to 24 months, and 2–8‐year intervals. Similarly, there were significant inverse associations between age at menarche and growth in height from the 2–3‐year interval onwards and in the birth to 13 months, birth to 24 months and 2–8‐year intervals. The strongest association in a year‐long interval for both >M1 and menarche was observed in the 6–7‐year interval. A 1 SD increase in height during the 6–7‐year interval corresponded with 3.0 months (= 0.25 years, CI 0.15 to 0.34, *p*‐value < 0.001) earlier age at >M1 and with 2.2 months (= 0.18 years, CI 0.08 to 0.28, *p*‐value < 0.001) earlier age at menarche. A direct association between height development and pubertal duration was significant in the 13–24‐month interval and from the 3–4‐year interval onwards and in the 2–8‐year interval (Table S3).

### 3.2 Boys

Weight gain and age at T ≥ 25 mm were inversely associated in four non‐consecutive age intervals: birth to 7 months, 13–24 months, 3–4 years and 7–8 years. In addition to the intervals describing infancy growth (birth to 13 months, birth to 24 months) and childhood growth (2–8 years) (Table 3), the strongest association for weight gain was found in the 3–4‐year age interval, where a 1 SD increase in weight gain corresponded with a 2.5 months (= 0.21 years, CI 0.08 to 0.36, *p*‐value 0.002) earlier age at T ≥ 25 mm. Following a similar pattern, weight gain was inversely associated with the age of G5 in four non‐consecutive age intervals: birth to 7 months, 3–4 years, 5–6 years and 7–8 years and similarly to the patterns describing infancy and childhood growth. The strongest association was found for weight gain in the 3–4‐year age interval, where a 1 SD increase in weight gain was associated with 2.2 months (= 0.18 years, CI 0.06 to 0.30, *p*‐value 0.003) earlier age at G5. Contrary to girls, there were no associations between weight gain and duration of puberty in boys.

**TABLE 3:** Associations of weight gain (standard deviation score, *z*‐score) with youngest age of testes length ≥ 25 mm, age of Tanner G5 and duration of puberty in boys.

	Age at testes length ≥ 25 mm (years)	*p*	Age at Tanner G5 (years)	*p*	Duration of Puberty^a^ (years)	*p*
	Beta^b^ CI		Beta^b^ CI		Beta^b^ CI	
Birth to 7 months	**−0.17** **−0.30 to − 0.04**	**0.01**	**−0.17** **−0.29 to − 0.05**	**0.004**	−0.04 −0.15 to 0.07	0.46
7 to 13 months	−0.01 −0.14 to 0.11	0.83	−0.07 −0.19 to 0.05	0.22	−0.05 −0.16 to 0.06	0.35
13 to 18 months	−0.12 −0.26 to 0.02	0.09	−0.04 −0.18 to 0.09	0.54	0.06 −0.6 to 0.18	0.35
18 to 24 months	−0.10 −0.24 to 0.04	0.15	0.02 −0.11 to 0.15	0.75	0.09 −0.03 to 0.20	0.16
Birth to 13 months	**−0.15** **−0.27 to − 0.02**	**0.02**	**−0.17** **−0.29 to − 0.06**	**0.003**	−0.05 −0.16 to 0.05	0.32
Birth to 24 months	**−0.19** **−0.32 to − 0.06**	**0.004**	**−0.14** **−0.26 to − 0.02**	**0.02**	−0.0002 −0.11 to 0.11	0.99
13 to 24 months	**−0.16** **−0.29 to − 0.02**	**0.02**	−0.003 −0.13 to 0.12	0.95	0.10 −0.13 to 0.21	0.08
2 to 3 years	0.01 −0.13 to 0.15	0.89	−0.02 −0.15 to 0.10	0.73	−0.02 −0.14 to 0.09	0.70
3 to 4 years	**−0.21** **−0.36 to − 0.08**	**0.002**	**−0.18** **−0.30 to − 0.06**	**0.003**	−0.004 −0.12 to 0.11	0.94
4 to 5 years	−0.08 −0.21 to 0.05	0.25	−0.08 −0.20 to 0.04	0.18	−0.0001 −0.11 to 0.11	0.99
5 to 6 years	−0.12 −0.25 to 0.01	0.08	**−0.14** **−0.26 to − 0.01**	**0.03**	−0.01 −0.12 to 0.10	0.85
6 to 7 years	−0.03 −0.16 to 0.10	0.67	−0.04 −0.16 to 0.08	0.54	−0.02 −0.13 to 0.10	0.78
7 to 8 years	**−0.20** **−0.33 to − 0.07**	**0.003**	**−0.14** **−0.27 to − 0.02**	**0.02**	0.03 −0.09 to 0.14	0.62
2 to 8 years	**−0.18** **−0.32 to − 0.05**	**0.01**	**−0.17** **−0.29 to − 0.04**	**0.008**	−0.01 −0.12 to 0.11	0.93

*Note:* Bold indicates *p* < 0.05.

^a^ Testis length ≥ 25 mm to Tanner G5 in boys.

^b^ The beta estimate represents the association of 1 SD increase in growth with the outcome variable in years (Beta × 12 gives the estimate in months). A negative value describes an earlier pubertal milestone/shorter duration of puberty.

When assessing growth using height development, the results were in line with the analysis of weight gain. An inverse association between height development and age at T ≥ 25 mm was significant in the birth to 13 months, 2–3 years and 4–5‐year intervals, and in the 2‐ to 8‐year interval. The strongest association in a year‐long interval for T ≥ 25 mm was observed in the 2–3‐year interval. A 1 SD increase in height during this interval corresponded with 2.8 months (= 0.23 years, CI 0.10 to 0.37, *p*‐value < 0.001) earlier age at T ≥ 25 mm. For the age of G5, inverse associations were found for height development in the 7–13 month, 13–18 month, 13–24 month, 2–3 year, 7–8 year and 2–8‐year intervals. The strongest association in a year‐long interval for G5 was observed in the 7–8‐year interval. A 1 SD increase in height during this interval corresponded with 2.6 months (= 0.22 years, CI 0.09 to 0.35, *p*‐value < 0.001) earlier age at G5. There was no association between growth in height and pubertal duration in boys (Table S4).

### 3.3 Adjusting for BMI

When adjusting weight gain analyses for BMI, the results remained essentially similar for both girls and boys (data not shown).

## 4 Discussion

The findings in this study strengthen previous observations of the association between early growth and pubertal development. We found that greater weight gain from birth to 2 years of age and during childhood growth (2–8 years) predicted a 5.2 months earlier onset of breast development and 3.6 months earlier menarche, with a 2.5‐months shorter duration of puberty per 1SD increase in weight gain [10, 25]. Likewise, in the same period of growth, each 1SD increase in height predicted a 4.7 months earlier onset of breast development and 3.1 months earlier menarche, with a 2.4 months shorter duration of puberty. Thus, our results further verify the trend of lengthening pubertal duration for girls as thelarche occurs at a younger age, as the age of menarche seems to remain stable [26, 27]. As in previous studies, we found that the association between early growth and pubertal development in boys is less evident compared to girls. In the longest growth interval during childhood growth (2–8 years of age) a 1 SD increase in weight gain and height advanced the start and culmination of genital development in boys by about 2 months. Similar to a previous report, our data showed that growth during the first year and the first 2 years of life was associated with an earlier age of pubertal development in both boys and girls [11]. Further, our results indicated childhood growth to be more significant than infancy growth in predicting pubertal development and that some intervals during childhood growth may have more impact than others.

During the last few decades, pubertal development has been observed to start earlier, and childhood obesity has been proposed as one of many possible factors [7, 28]. Known risk factors for early or precocious puberty, such as intrauterine growth restriction or being born small for gestational age, are well established, as is catch‐up growth in infancy [29]. Our study showed that weight gain in infancy associates with pubertal timing even in normal weight children and in those with birth weight AGA. Early puberty has been associated with an increased risk for cardiometabolic diseases, such as increased BMI, unfavourable blood lipids and glucose metabolism, in addition to cancer and psychosocial difficulties [3, 4, 5, 30]. Although associations between early weight gain and pubertal timing and tempo were rather modest, we think that identifying patterns that may predict earlier onset of puberty, such as increased weight gain in childhood, may provide information to improve public health by targeting specific individuals.

### 4.1 Strengths and Limitations

The strengths of the study were the well‐established, meticulous methods applied in STRIP among a large number of frequently and repeatedly studied participants in a prospective setting. Additionally, most participants were AGA and normal weight throughout the study period, enabling us to investigate the impact of growth in a seemingly ‘normally’ growing population. The short age intervals before 2 years of age were possible because of the frequent study visits at the beginning of the STRIP. Later, the annual study visits provided steady follow‐ups of pubertal development and growth. A significant strength is that pubertal development was clinically evaluated with proper measurements and not self‐reported.

A potential limitation was the loss to follow up that occurs in every longitudinal study – especially those that last as long as STRIP. However, no significant differences between those who continued in the study and those who were lost to follow‐up have been found [19]. Thus, this should not cause a systematic selection bias [19]. A limitation is that only the intervention group had study visits twice a year while the control group had study visits only once a year; consequently, evaluations of pubertal development included in this study were only done annually for 50% of the participants. This is why we chose to include the first yearly measurement surpassing M1/T ≥ 25 mm as the start of puberty. Additionally, a limitation is that assessment of pubertal development began at 9 years of age, as normal pubertal development for girls may begin already at 8 years of age. Age of menarche was self‐reported at the annual visit, which may cause some bias in the analyses. However, most participants were able to report the beginning of menstruation with an accuracy of 1 month. Possible inaccuracy in reporting the age of menarche was accounted for by excluding those girls whose puberty duration was < 0 year, in contrast to < 1 year as would be considered normal pubertal duration. Information on parents' pubertal development was not available, and neither was the duration of breastfeeding. Lastly, the participants in the STRIP were Caucasian; thus, the results may not be generalisable to other ethnicities.

## 5 Conclusion

Increased infancy and childhood growth were associated with earlier age at pubertal milestones in both boys and girls, and with a longer duration of puberty in girls but not in boys. The findings in this study increase the understanding of the associations between pubertal development and growth in infancy and childhood. This could help identify individuals who may be predisposed to normal puberty at an earlier age and who risk possible associated health concerns later in life.

## Conflicts of Interest

The authors declare no conflicts of interest.

## Supporting information


Tables S1–S4.

## Data Availability

Research data are not shared.
