# Supplementary material for: Weight Gain in Infancy and Childhood Were Associated With Pubertal Development in Boys and Girls
Source: Acta Paediatr. 2025 May 23;114(10):2612–8. doi: 10.1111/apa.70148 (PMC12420895; doi:10.1111/apa.70148)
Supplement: Supplementary file 1 — Tables S1–S4. [file APA-114-2612-s001.docx]

**Supplementary Tables**

**Table S1. Weight gain in varying age intervals among girls and boys.**

|  | **Weight gain (kg)** | | | |
| --- | --- | --- | --- | --- |
|  | **Girls** | | **Boys** | |
| **Age interval** | n | Mean, (SD) | n | Mean, (SD) |
| Birth to 7 months | 275 | 4.70 (0.81) | 229 | 5.13 (0.74) |
| 7 to 13 months | 275 | 1.78 (0.58) | 229 | 1.79 (0.52) |
| 13 to 18 months | 262 | 1.33 (0.51) | 214 | 1.30 (0.49) |
| 18 to 24 months | 253 | 1.37 (0.59) | 211 | 1.20 (0.48) |
| Birth to 13 months | 278 | 6.49 (0.94) | 230 | 6.94 (0.89) |
| Birth to 24 months | 269 | 9.18 (1.38) | 223 | 9.42 (1.15) |
| 13 to 24 months | 269 | 2.68 (0.83) | 223 | 2.48 (0.68) |
| 2 to 3 years | 258 | 2.26 (0.76) | 221 | 2.18 (0.66) |
| 3 to 4 years | 264 | 2.22 (0.80) | 225 | 2.06 (0.67) |
| 4 to 5 years | 268 | 2.19 (0.95) | 225 | 1.99 (0.75) |
| 5 to 6 years | 268 | 2.83 (1.14) | 227 | 2.67 (1.09) |
| 6 to 7 years | 263 | 2.80 (1.42) | 222 | 2.56 (1.13) |
| 7 to 8 years | 265 | 3.29 (1.52) | 213 | 3.10 (1.18) |
| 2 to 8 years | 263 | 15.55 (4.42) | 211 | 14.52 (3.27) |

**Table S2. Height development in varying age intervals among girls and boys.**

|  | **Weight gain (cm)** | | | |
| --- | --- | --- | --- | --- |
|  | **Girls** | | **Boys** | |
| **Age interval** | n | Mean, (SD) | n | Mean, (SD) |
| Birth to 7 months | 274 | 19.31 (2.18) | 229 | 19.87 (2.11) |
| 7 to 13 months | 274 | 7.57 (1.61) | 229 | 7.49 (1.52) |
| 13 to 18 months | 259 | 5.66 (1.50) | 213 | 5.62 (1.47) |
| 18 to 24 months | 248 | 5.10 (1.47) | 209 | 4.66 (1.41) |
| Birth to 13 months | 278 | 26.92 (2.45) | 230 | 27.38 (2.78) |
| Birth to 24 months | 267 | 37.65 (2.73) | 222 | 37.65 (2.46) |
| 13 to 24 months | 267 | 10.67 (1.62) | 222 | 10.28 (1.56) |
| 2 to 3 years | 255 | 8.75 (1.52) | 219 | 8.57 (1.43) |
| 3 to 4 years | 263 | 7.68 (1.18) | 224 | 7.49 (1.05) |
| 4 to 5 years | 268 | 7.43 (0.92) | 225 | 7.42 (0.93) |
| 5 to 6 years | 267 | 6.08 (1.00) | 227 | 6.05 (0.92) |
| 6 to 7 years | 262 | 6.65 (0.99) | 222 | 6.67 (0.92) |
| 7 to 8 years | 265 | 5.29 (0.98) | 213 | 5.19 (0.95) |
| 2 to 8 years | 261 | 41.93 (3.54) | 210 | 41.34 (3.26) |

**Table S3. Associations of height development (standard deviation score, z-score) with youngest age of Tanner stage >M1, age of menarche, and duration of puberty in girls.**

|  | **Age at Tanner >M1**  **(years)** |  | **Age at menarche**  **(years)** |  | **Duration of Puberty^a^**  **(years)** |  |
| --- | --- | --- | --- | --- | --- | --- |
|  | Beta*  CI | p-value | Beta*  CI | p-value | Beta*  CI | p-value |
| Birth to 7 months | **-0.10**  **-0.19 to -0.003** | **0.04** | **-0.12**  **-0.21 to 0.02** | **0.02** | -0.01  -0.12 to 0.10 | 0.85 |
| 7 to 13 months | 0.04  -0.05 to 0.13 | 0.39 | -0.02  -0.12 to 0.08 | 0.70 | -0.08  -0.18 to 0.03 | 0.16 |
| 13 to 18 months | **-0.10**  **-0.20 to -0.01** | **0.03** | -0.06  -0.16 to 0.05 | 0.28 | 0.08  -0.04 to 0.19 | 0.18 |
| 18 to 24 months | -0.06  -0.16 to 0.04 | 0.24 | 0.03  -0.08 to 0.13 | 0.64 | 0.11  -0.004 to 0.22 | 0.06 |
| Birth to 13 months | -0.07  -0.16 to 0.02 | 0.13 | **-0.13**  **-0.23 to -0.03** | **0.01** | -0.06  -0.17 to 0.05 | 0.27 |
| Birth to 24 months | **-0.16**  **-0.26 to -0.07** | **0.001** | **-0.16**  **-0.26 to -0.06** | **0.003** | 0.03  -0.08 to 0.15 | 0.57 |
| 13 to 24 months | **-0.16**  **-0.25 to -0.07** | **0.001** | -0.06  -0.16 to -0.03 | 0.19 | **0.14**  **0.03 to 0.24** | **0.01** |
| 2 to 3 years | **-0.15**  **-0.24 to -0.07** | **<0.001** | **-0.13**  **-0.23 to -0.03** | **0.01** | 0.05  -0.06 to 0.16 | 0.41 |
| 3 to 4 years | **-0.22**  **-0.31 to -0.13** | **<0.001** | **-0.14**  **-0.24 to -0.04** | **0.01** | **0.13**  **0.02 to 0.24** | **0.02** |
| 4 to 5 years | **-0.23**  **-0.31 to -0.14** | **<0.001** | **-0.14**  **-0.23 to -0.04** | **0.01** | **0.14**  **0.04 to 0.25** | **0.01** |
| 5 to 6 years | **-0.25**  **-0.34 to -0.16** | **<0.001** | **-0.13**  **-0.23 to -0.03** | **0.01** | **0.17**  **0.06 to 0.28** | **0.002** |
| 6 to 7 years | **-0.25**  **-0.34 to -0.15** | **<0.001** | **-0.18**  **-0.28 to -0.08** | **<0.001** | **0.11**  **-0.01 to 0.22** | **0.07** |
| 7 to 8 years | **-0.23**  **-0.32 to -0.14** | **<0.001** | **-0.16**  **-0.26 to -0.07** | **<0.001** | **0.11**  **0.001 to 0.22** | **0.048** |
| 2 to 8 years | **-0.39**  **-0.47 to -0.31** | **<0.001** | **-0.26**  **-0.36 to -0.17** | **<0.001** | **0.20**  **0.10 to 0.31** | **<0.001** |

^a^ >M1 to Menarche in girls
*the beta estimate represents association of 1 SD increase in growth with the outcome variable in years (Beta x 12 gives the estimate in months). A negative value describes earlier pubertal milestone/shorter duration of puberty.
Bold indicates p < 0.05

**Table S4. Associations of height development (standard deviation score, z-score) with youngest age of testes length ≥25 mm, age of Tanner G5, and duration of puberty in boys.**

|  | **Age at testes ≥25 mm**  **(years)** |  | **Age at Tanner G5**  **(years)** |  | **Duration of Puberty^a^**  **(years)** |  |
| --- | --- | --- | --- | --- | --- | --- |
|  | Beta*  CI | p-value | Beta*  CI | p-value | Beta*  CI | p-value |
| Birth to 7 months | -0.08  -0.21 to 0.05 | 0.24 | -0.01  -0.14 to 0.11 | 0.83 | 0.03  -0.08 to 0.14 | 0.55 |
| 7 to 13 months | -0.07  -0.20 to 0.06 | 0.29 | **-0.15**  **-0.27 to -0.03** | **0.02** | -0.08  -0.18 to 0.03 | 0.17 |
| 13 to 18 months | -0.01  -0.15 to 0.13 | 0.88 | **0.14**  **0.01 to 0.28** | **0.03** | 0.12  -0.0004 to 0.24 | 0.051 |
| 18 to 24 months | 0.04  -0.10 to 0.18 | 0.57 | 0.02  -0.12 to 0.15 | 0.80 | -0.01  -0.13 to 0.11 | 0.85 |
| Birth to 13 months | **-0.12**  **-0.25 to 0.01** | **0.07** | -0.10  -0.23 to 0.02 | 0.09 | -0.01  -0.12 to 0.10 | 0.81 |
| Birth to 24 months | -0.12  -0.25 to 0.02 | 0.08 | -0.03  -0.16 to 0.09 | 0.58 | 0.04  -0.07 to 0.15 | 0.51 |
| 13 to 24 months | 0.01  -0.12 to 0.15 | 0.83 | **0.13**  **0.01 to 0.25** | **0.04** | 0.09  -0.02 to 0.20 | 0.10 |
| 2 to 3 years | **-0.23**  **-0.37 to -0.10** | **<0.001** | **-0.16**  **-0.29 to -0.04** | **0.01** | 0.02  -0.09 to 0.14 | 0.70 |
| 3 to 4 years | -0.10  -0.23 to 0.03 | 0.14 | -0.09  -0.22 to 0.03 | 0.14 | -0.01  -0.13 to 0.10 | 0.85 |
| 4 to 5 years | **-0.15**  **-0.29 to -0.02** | **0.02** | -0.09  -0.22 to 0.03 | 0.13 | 0.04  -0.08 to 0.15 | 0.53 |
| 5 to 6 years | -0.05  -0.18 to 0.08 | 0.44 | -0.03  -0.15 to 0.10 | 0.67 | 0.03  -0.08 to 0.14 | 0.62 |
| 6 to 7 years | -0.04  -0.18 to 0.09 | 0.52 | -0.10  -0.23 to 0.02 | 0.11 | -0.06  -0.18 to 0.05 | 0.28 |
| 7 to 8 years | -0.14  -0.27 to 0.002 | 0.053 | **-0.22**  **-0.35 to -0.09** | **<0.001** | -0.07  -0.19 to 0.05 | 0.26 |
| 2 to 8 years | **-0.23**  **-0.37 to -0.09** | **0.002** | **-0.21**  **-0.34 to -0.08** | **0.002** | -0.01  -0.13 to 0.11 | 0.90 |

^a^ testes ≥25 mm to Tanner G5 in boys
*the beta estimate represents association of 1 SD increase in growth with the outcome variable in years (Beta x 12 gives the estimate in months). A negative value describes earlier pubertal milestone/shorter duration of puberty.
Bold indicates p < 0.05
